# Quantitative determination of free D-Asp, L-Asp and N-methyl-D-aspartate in mouse brain tissues by chiral separation and Multiple Reaction Monitoring tandem mass spectrometry

**DOI:** 10.1371/journal.pone.0179748

**Published:** 2017-06-29

**Authors:** Carolina Fontanarosa, Francesca Pane, Nunzio Sepe, Gabriella Pinto, Marco Trifuoggi, Marta Squillace, Francesco Errico, Alessandro Usiello, Piero Pucci, Angela Amoresano

**Affiliations:** 1 Department of Chemical Sciences, University of Naples “Federico II”, Naples, Italy; 2 Istituto Nazionale Biostrutture e Biosistemi, Roma, Italy; 3 CEINGE Advanced Biotechnology, University of Naples “Federico II”, Naples, Italy; 4 Department of Molecular Medicine and Medical Biotechnology, University of Naples “Federico II”, Naples, Italy; Indian Institute of Chemical Technology, INDIA

## Abstract

Several studies have suggested that free d-Asp has a crucial role in N-methyl d-Asp receptor-mediated neurotransmission playing very important functions in physiological and pathological processes. This paper describes the development of an analytical procedure for the direct and simultaneous determination of free d-Asp, l-Asp and N-methyl d-Asp in specimens of different mouse brain tissues using chiral LC-MS/MS in Multiple Reaction Monitoring scan mode. After comparing three procedures and different buffers and extraction solvents, a simple preparation procedure was selected the analytes of extraction. The method was validated by analyzing l-Asp, d-Asp and N-methyl d-Asp recovery at different spiked concentrations (50, 100 and 200 pg/μl) yielding satisfactory recoveries (75–110%), and good repeatability. Limits of detection (LOD) resulted to be 0.52 pg/μl for d-Asp, 0.46 pg/μl for l-Asp and 0.54 pg/μl for NMDA, respectively. Limits of quantification (LOQ) were 1.57 pg/μl for d-Asp, 1.41 pg/μl for l-Asp and 1.64 pg/μl for NMDA, respectively. Different concentration levels were used for constructing the calibration curves which showed good linearity. The validated method was then successfully applied to the simultaneous detection of d-Asp, l-Asp and NMDA in mouse brain tissues. The concurrent, sensitive, fast, and reproducible measurement of these metabolites in brain tissues will be useful to correlate the amount of free d-Asp with relevant neurological processes, making the LC-MS/MS MRM method well suited, not only for research work but also for clinical analyses.

## Introduction

Although the l-enantiomer forms of amino acids greatly predominate in nature, d-enantiomers generated from the post-translational isomerization modifications on l-amino acids have been detected in bacteria, plants, in different phyla of invertebrates as well as in vertebrate animals up to mammals, including humans [1–5]. Their appeal derives from the multiplicity of performed physiological functions, some of them not yet elucidated. d-Ala, d-Glu, d-Asn are components of bacteria cell wall peptidoglycans and in the same organisms d-Phe, d-Tyr, d-Met and d-Leu appeared to modulate peptidoglycans synthesis displaying a regulatory mechanism of cellular growth [6]. The slight structural changes due to the chirality inversion seem to be responsible for the bioactivity of d-amino acid containing peptides as it occurs for dermorphin where d-Ala displays selectivity and high affinity for human μ-type opiate receptors [7]. Among all the d-amino acids, d-Ser and d-Asp are the amino acids more investigated for their crucial role especially in mammalian tissues [8–9]. Indeed, the concentration of d-Asp follows a peculiar temporal pattern of emergence in endocrine glands, with its increase in the postnatal and adult phase following the functional maturation of these organs [10]. Conversely, in the brain, the strong decrease of free d-Asp levels after birth is a consequence of the postnatal onset of d-aspartate oxidase (DDO), the only known enzyme that catabolizes d-Asp [11]. In this organ, d-Asp appeared particularly concentrated in the synaptic vesicles of terminal axon, suggesting its role as an endogenous neurotransmitter [10, 12]. d-Asp enhances hippocampal N-Methyl d-Asp receptor (NMDAR)-dependent synaptic plasticity, dendritic morphology, spatial memory and learning processes [13]. A significant reduction of d-Asp levels has been also recently found in the prefrontal cortex of patients with schizophrenia, associated with an increased expression of DDO mRNA [14].

Due to its physiological relevance, a number of different methods have been proposed to detect d-Asp, including gas chromatography, high-performance liquid chromatography (HPLC), high-performance capillary electrophoresis (CE) and enzymatic procedures [15–16]. Besides the enantioseparation, the analysis of d-Asp in biological samples is challenging because of the large amount of its l-counterpart and the presence of several biological substances interfering with the analysis. However, the crucial issue correlated to this analysis remains the enantioseparation of d-Asp from l-Asp, due to their similar chemical and physical properties, excluding configuration.

Many papers describe protocols including a preliminary step of derivatisation with a fluorogenic chiral reagent [17] or with fluorogenic non-chiral reagents providing a separation on single chiral stationary phase [18] or on two subsequent chromatographic steps linked by an automated column-switching device [19–24]. Other methods provide the conversion of each enantiomer into octyl ester derivatives for the direct gas chromatography-mass spectrometry (GC-MS) analysis, as described for the determination of N-methyl-dl-aspartic acid (NMA) [25]. However, most of enantionseparation protocols are based on coupling of liquid chromatography to mass spectrometry as that combining Chirobiotic T chiral stationary phase column to atmospheric pressure chemical ionization (APCI) mass spectrometry for the quantification of 25 amino acid enantiomers [26]. Others are focused on the optimization of performing MS analysis e.g. by exploiting the different level of vibrational excitation of enantiomers in two different dissociation modes [27], or on chiral separation by ion mobility spectrometry [28].

Targeted metabolomics making use of LC-MS/MS in Multiple Reaction Monitoring (MRM) scan mode was shown to be effective in identifying altered metabolic pathways in pathological conditions and/or to define therapeutic modes of action [29]. In this context, recently Livnat and co-workers successfully separated all the twenty l-amino acids from their isomers on Phenyl-Hexyl column and quantified them in a single MRM run [4]. However, this protocol suffered of longer preliminary steps of derivatisation of the l- and D-amino acids with the Marfey’s reagent.

The present paper describes the development of a simple and reliable procedure for the extraction and the simultaneous direct determination of free l-Asp, d-Asp, and its derivative NMDA in specimens of mouse brain tissues by coupling chiral liquid chromatography to tandem mass spectrometry in MRM scan mode. Given the crucial role of DDO enzyme in the regulation of d-Asp physiological levels, the above mentioned metabolites were successful extracted by brain tissues from wild-type (*DDO*^+/+^) and knockout (*DDO*^-/-^) mice, separated by HPLC Astec Chirobiotic chiral phase column and quantified by single run LC-MS/MS-MRM analysis without any derivatisation step. This preliminary study is addressed at the specific, sensitive, fast and reproducible measurement of this metabolites in brain tissues in order to correlate the amount of d-Asp with relevant neurodegenerative processes and to monitor the effect of specific therapeutic treatments.

Finally, the developed method has the great potentiality to be extended to a wide number of molecules, including all the existing d-amino acids, and the possibility to simultaneously monitor them in a single run, by optimization of the MRM parameters. This approach could open new insights into the clinical diagnostics of neuropathological diseases.

## Materials and methods

### Chemicals and reagents

N-methyl d-Asp (NMDA), d-Asp, l-Asp, were purchased from Sigma-Aldrich. All solutions and solvents of the highest available purity and suitable for LC-MS analysis were purchased from J. T. Baker (Phillipsburg, NJ). All stock solutions were stored at −20°C.

All research involving animals was performed in accordance with the European directive 86/609/EEC governing animal welfare and protection, and was approved by the Animal Welfare and Ethical Review Body Committee (AWERBC) of Ceinge Biotecnologie Avanzate in the light of the project no GR-2009-1605759 approved by the Italian Ministry of Health.

Knockout mice for the *DDO* gene were generated and genotyped by PCR as described previously [30]. Wild-type (*DDO*^+/+^) and knockout (*DDO*^-/-^) mice were housed in groups (n = 4–5) in standard cages (29 x 17.5 x 12.5 cm) at constant temperature (22 ± 1°C) and maintained on a 12/12 h light/dark cycle, with food and water ad libitum. All efforts were made to minimize the animal suffering. Mice were killed by decapitation and the prefrontal cortex dissected out within 20 s on an ice-cold surface and then stored at -80°C for subsequent analyses.

### Preparation of standard solutions

Stock solutions were prepared by adding 1.0 mL aliquots of each analyte to a 10-mL volumetric flask and bringing the standard to volume with methanol to yield a standard solution with 1000 ng/mL of each analyte. Stock solutions were stored at −20°C until the analysis.

### Individual standard solution

Final 2000 pg/μl individual analyte standard solutions were prepared by serial dilution from stock solutions and used for calibration curves. Standard mixtures for each analyte were prepared at the following concentrations: 0.5, 1, 25, 50, 125, 200, 300 pg/μl for d-Asp; 1, 5, 25, 50, 125 pg/μl for l-Asp; 0.25, 0.5, 2.5, 5, 10, 25, 50 for NMDA. All standards were kept at -20°C before LC-MS/MS analysis.

### Sample preparation

Aliquots of 0.5 g of mouse brain tissues were homogenized using different buffers including ammonium bicarbonate 10 mM pH7.5, ammonium bicarbonate 50 mM pH 8.0, Tris-HCl 50 mM pH 8.0, Hepes 50mM pH 8.0. Samples were added with 100 pg/μl of standard analytes to quantitatively evaluate the best conditions of sample preparation. Samples were then sonicated for different times (from 10 to 120 min with 10 min increments) until a clear solution was obtained. Each test sample was then divided in three aliquots and the analytes extracted in the supernatant by precipitation of the protein components using different solvent systems (acetonitrile, ethanol, methanol). An aliquot of 0.6 ml of organic solvent was added to each sample and the tube was mechanically shaken vigorously for 5 min. The tube was centrifuged at 10000 rpm (7600 rcf) at 10°C for 10 min. Using a Pasteur pipette, the upper organic layer was transferred into a centrifuge tube. 0.2 ml of upper organic layer was filtered through a 0.2-μm PTFE syringe filter (Pall Acrodisc 13 mm) into an LC vial for analysis.

Once defined the best conditions for sample preparation, control samples were fortified in triplicate at three different target levels (50, 100 and 200 pg/μl) by adding the standard solution containing the same concentration of the three analytes to 200 μl of homogenized control tissues (0.5 g) for validation of the method.

### LC–MS/MS instrumentation and conditions

The supernatant (2 μl) were analysed by using an Agilent 6420 Triple Quadrupole LC-MS/MS system with a HPLC 1100 series binary pump (Agilent, Waldbronn, Germany).

A mixture of d-Asp, l-Asp and NMDA (100 pg/μl each) was resolved on a HPLC Astec Chirobiotic chiral phase column (10 cm x 4.6mm, 5μm), consisting of the amphoteric glycopeptide Teicoplanin covalently bound to a 5 μm spherical silica gel through multiple covalent linkages. The mobile phase was generated by mixing eluent A (0.1% Formic Acid in 2% ACN) and eluent B (0.009% Formic Acid in methanol) and the flow rate was 0.5 mL/min. Elution gradient was from 50% to 95% B in 6 min.

Tandem mass spectrometry was performed using a turbo ion spray source operated in positive mode, and the MRM mode was used for the selected analytes. A standard solution of 500 pg/μl of each metabolite was used for optimization of the MRM transition as reported in Table 1. Metabolites were automatically (flow injection) tuned for ionization polarity, optimal declustering potential (DP), product ion, and collision energy (CE) using metabolite standard solutions via Agilent MassHunter Optimizer software. Table 1 provides a list of precursor ion, product ions, collision energy and retention times for all analytes.

**Table 1:** Mass spectral parameters and retention times for the l-Asp, d-Asp and NMDA analytes. The most intense transitions for each analyte, 134.0>116.0 for l-Asp and d-Asp and 148.0>88.0 for NMDA were used for the quantification while the others were used for the identification.

Compound Name	Precursor Ion (m/z)	Product Ion (m/z)	Dwell (ms)	Abundance (counts)	Fragmentor (V)	Collision Energy (V)	Cell Accelerator Voltage (V)	Polarity	RT (min)
l-Asp	134.0	116.0	200	15478	81	1	7	Positive	3.38
	134.0	88.0	200	12365	81	8	7	Positive	
	134.0	74.0	200	10115	81	9	7	Positive	
D-Asp	134.0	116.0	200	13254	81	1	7	Positive	3.73
	134.0	88.0	200	12356	81	8	7	Positive	
	134.0	74.0	200	10365	81	9	7	Positive	
NMDA	148.0	130.0	200	874	81	1	7	Positive	4.4
	148.0	88.0	200	16485	81	9	7	Positive	
	148.0	42.0	200	12474	81	25	7	Positive	

### Data processing

Extracted mass chromatogram peaks of metabolites were integrated using Agilent MassHunter Quantitative Analysis software (B.05.00). Peak areas of corresponding metabolites are then used as quantitative measurements for assay performance assessments such as variation, linearity etc.

### Method validation

#### Limit of detection and quantitation

The limits of detection (LODs) were defined as the lower limit of concentration below which the sample could not be revealed and were determined by making 10 replicate measurements of blank samples spiked with low concentrations of each analyte. Calculations were made according to the following formula: LOD = 3 times standard deviation (SD).

The limit of quantitation (LOQ) is the lowest concentration at which the analyte can not only be reliably detected but also quantitated. The LOQ may be equivalent to the LOD or higher. Standard solutions were prepared by spiking known amount of d-Asp, l-Asp and NMDA in the control mouse brain sample. The metabolites were extracted and analysed by the LC-MS/MS procedure. LOQ was determined as the analyte concentration giving a signal to noise ratio (S/N) of 10.

#### Matrix effect

Possible matrix effects were evaluated by comparing standard and matrix-matched calibration curves for each analyte. Standard solutions were prepared as described. Wild type tissue samples treated with DDO were extracted as described and spiked with the target compounds, resulting in matrix-matched solutions. The calibration curves were repeated three times. Matrix effects were evaluated by comparing five points standard and matrix-matched calibration curves.

#### Specificity

The specificity of the assay was demonstrated by checking for interfering peaks at the retention time of the target analytes.

#### Stability

The stability of the analytes in matrices was determined on short-term conditions (−20°C, 7 days). All sample stability studies were evaluated by comparing the area of the analytes in freshly prepared matrices with that of storage matrices and performed in triplicate. When the area of the analytes in storage matrices was lower than 10% compared to the area of the analytes in freshly prepared matrices, the analytes in matrices were considered not stable.

#### Selectivity

Selectivity was evaluated by analyzing 20 blank samples. The blank samples were prepared by executing the whole analysis procedure without test sample and omitting the standards addition.

Selectivity was defined acceptable when no peaks were detected in the chromatogram of the procedural blank sample at the retention time of the analytes ± 0.1 minute or, if present, peaks did not exceed 30% of the height of the native analyte in the chromatogram of the lowest calibration level.

#### Linearity

The linearity of an analytical procedure is defined as its ability to obtain test results that are directly proportional to the concentration (amount) of analyte in the sample within a given range. Linearity was demonstrated directly on the standard solutions by dilution of a standard stock solution as described. Linearity was determined by a series of three injections of standards at different concentration levels. A linear regression equation was applied to the results. The linearity is evaluated graphically, by calibration curves, and by visually inspecting a plot of peak area as a function of analyte concentration

#### Precision (repeatability and intermediate precision)

Precision was assessed at the lowest limit of the working range level from matrix samples, which were spiked with a known amount of standard solutions Triplicate analyses on three different days were performed on each spiked sample.

Repeatability and intermediate precision estimates were calculated by ANOVA and expressed as relative standard deviation. The obtained repeatability relative standard deviations (RSDr) were calculated. Intermediate precision was evaluated from triplicate analysis in three independent analysis sequences performed over a period of one month.

#### Recovery

Recovery values were calculated from the measurement of standards spiked in test samples. The average recoveries and their relative standard deviations were calculated from all the results obtained for each standard via response factors, which were determined from calibration solutions as follows:

$$\text{Recovery}\left(\text{\%}\right)=\frac{{\text{c}}_{1}-{\text{c}}_{2}}{{\text{c}}_{3}}*100$$

where:

C1: analyte concentration measured after the addition

C2: analyte concentration measured before the addition

C3: added concentration

Recovery values were not used for correction of analysis results. They were only used to monitor the yield of the sample preparation procedures.

#### Measurement uncertainty

Measurement uncertainty was estimated according to the law of error propagation. The uncertainty contributions considered in the combined uncertainty were the uncertainties of the preparation of native standard solutions for instrument calibration, the uncertainty of the preparation of spiking solutions for the preparation of test samples, the uncertainty contribution deriving from instrument calibration, the uncertainty coming from the precision of the analyses and the uncertainty of bias.

## Results

Preliminary analyses were carried out for optimizing the HPLC separation and LC-MS/MS conditions by using l-Asp, d-Asp and NMDA as a standard metabolite solution. Several extraction procedures were also assessed and a validation process was followed for testing the matrix effect. LOD and LOQ, the precision and accuracy, the recovery in matrix before proceeding with the determination of d-Asp, l-Asp and NMDA in mouse brain tissues were also evaluated.

### Enantiomeric separation

**Fig 1:**
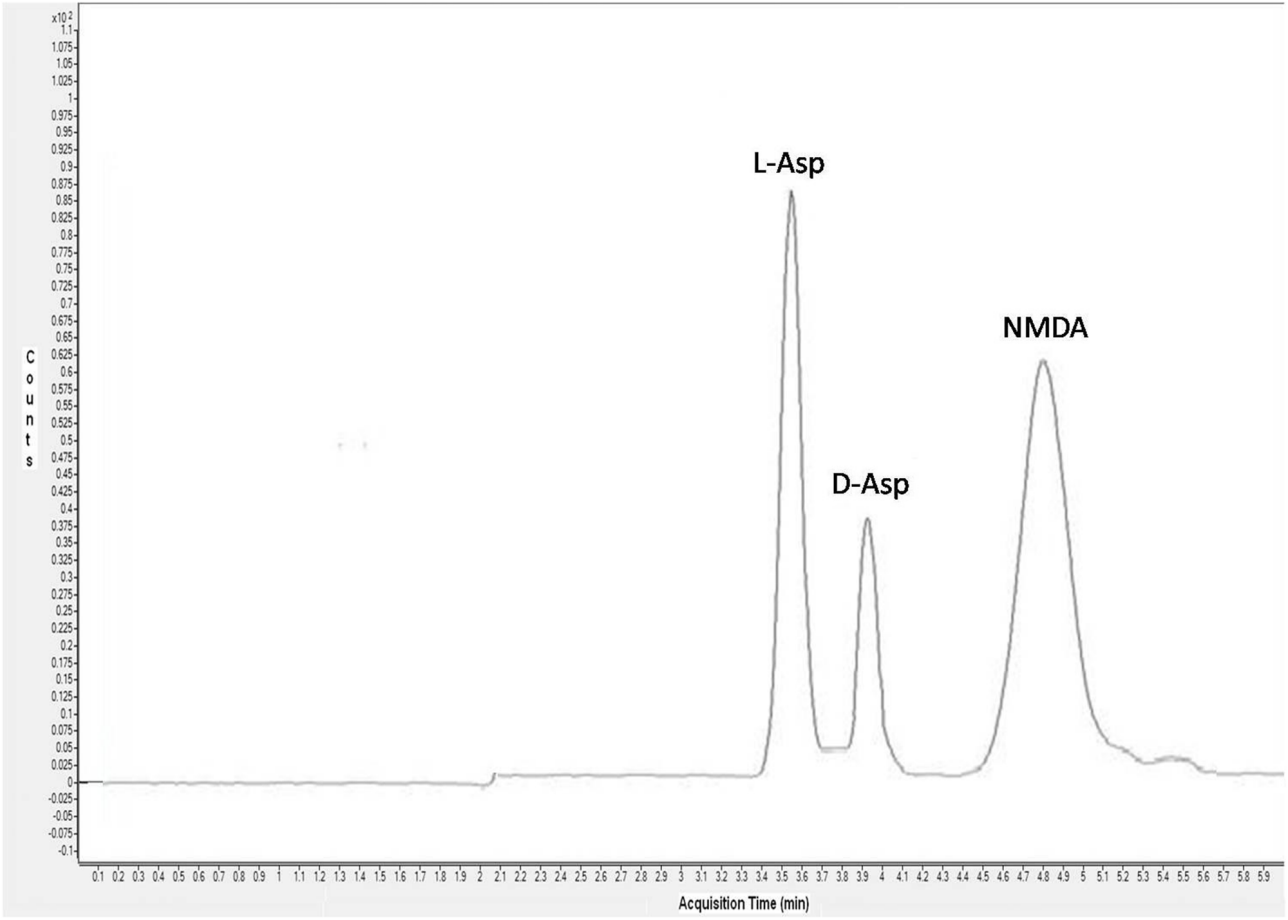
Total ion current (TIC) chromatogram for the analysis of 100 pg/μl standard mixture solution of l-Asp, d-Asp and NMDA. Peaks corresponding to each analyte are labelled.

The specific separation of the three analytes was obtained by using a Teicoplanin column containing 23 chiral centers surrounding four pockets or cavities with hydrogen donor and acceptor sites readily available [19]. This type of arrangement is known to be highly favourable for a number of enantiomeric separations. Elution of the enantiomers was accomplished by an unbuffered mixture of methanol and acetonitrile containing a small amount of formic acid providing an optimal coupling with the following mass spectral analysis. Fig 1 shows the total ion current (TIC) chromatogram obtained for the analysis of 100 pg/μl standard mixture solution showing a good peak shape for the three analytes. Several injections of the standard mixture were carried out to ensure reproducibility of retention times.

### Mass spectral parameters selection

Next step consisted in the selection of the best mass spectral parameters for the MRM detection of the analytes. d-Asp, l-Asp and NMDA were individually infused to establish the optimal instrument settings for each compound. Experimental automatic tuning by using MassHunter Optimizer was used to define ionization polarity, to select the best product ion (Q3 ion) and to optimize both the collision energy (CE) and the declustering potential (DP). The MRM transitions and all the instrumental parameters determined are summarised in Table 1. It should be underlined that the NMDA transitions were specific for this metabolite and different from its isomer Glu (148 → 130; 148 → 102; 148 → 84). The MRM chromatograms obtained for standard solutions of d-Asp, l-Asp and NMDA showing a good selectivity for all analytes, with all transitions correctly occurring at the same retention time are reported in S1 Fig.

### Sample extraction procedure

First, a reliable sample extraction procedure had to be optimised keeping sample manipulation to a minimum because of the low concentration of d-Asp occurring in most biological samples. Target analytes were extracted from freeze-dried specimens of mouse brain tissues following a two-steps procedure consisting in sonication of the sample and protein precipitation prior to LC-MS/MS analysis. Different buffers and sonication parameters were evaluated as reported below.

**Fig 2:**
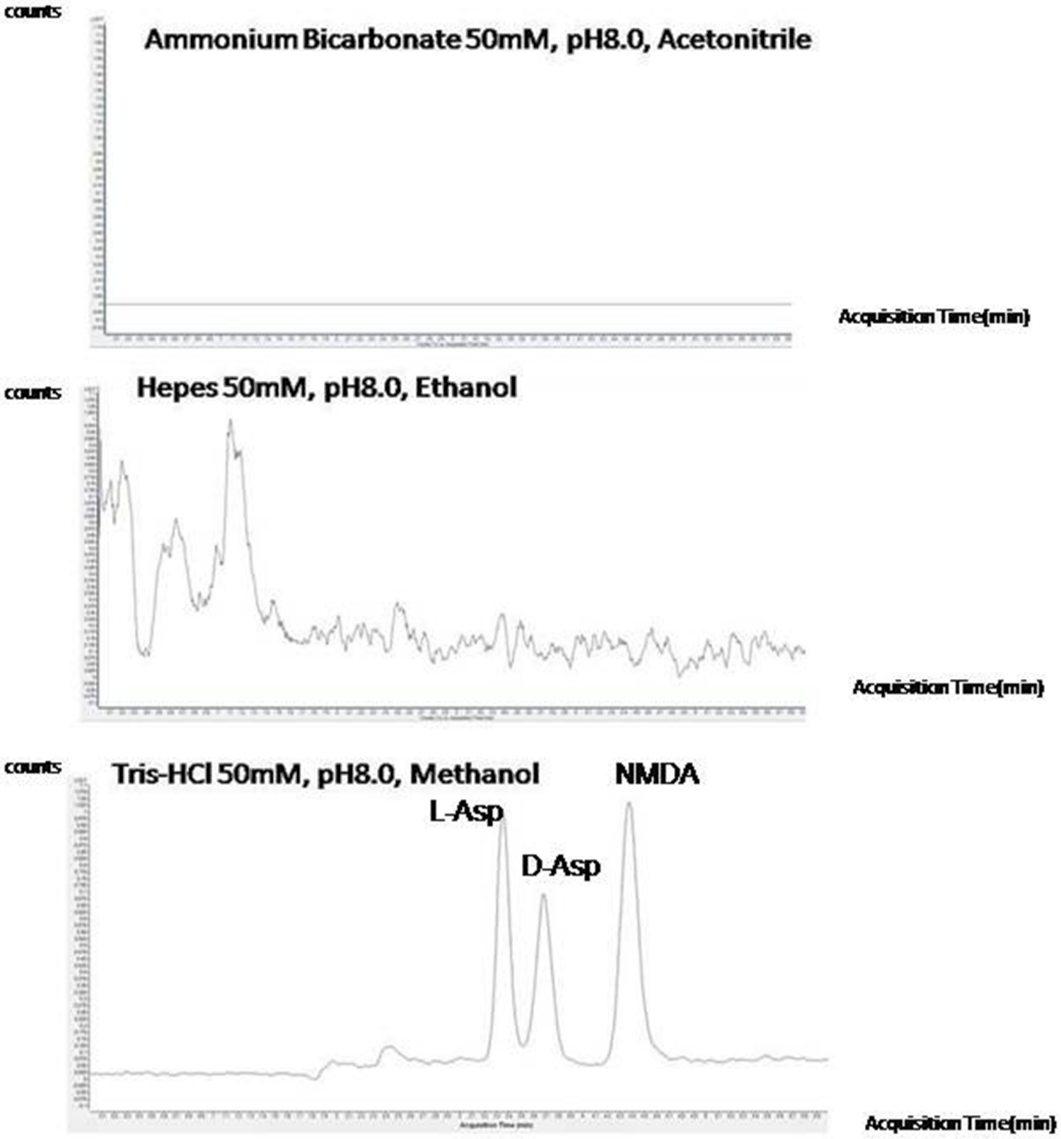
LCMSMS analyses in MRM mode of test samples from mouse brain tissues to optimise the sample treatment procedure. Some experimental conditions for sample extraction and precipitation are reported in the figure. Peaks corresponding to each analyte are labelled.

Each test sample was then divided in three aliquots and the analytes extracted in the supernatant by precipitation of the protein components using different solvent systems (acetonitrile, ethanol, methanol). All test samples were then analysed by the LC-MS/MS MRM procedure to evaluate the best conditions for sample preparation. As an example, Fig 2 shows the LC-MS/MS analyses in MRM mode of test samples from mouse brain tissues prepared in three different conditions. The recovery of analytes in the different conditions explored is reported in S1 Table. According to the data shown in S1 Table, the best results were obtained by dissolving the mouse brain samples in Tris-HCl 50 mM pH 8.0 followed by sonication for 10 min and using methanol for protein precipitation. Extending the precipitation time beyond 30 min (*i*.*e*., up to 2 hours) did not increase the analyte recovery. Experiments were performed in triplicate for each pair of conditions and averaged.

**Fig 3:**
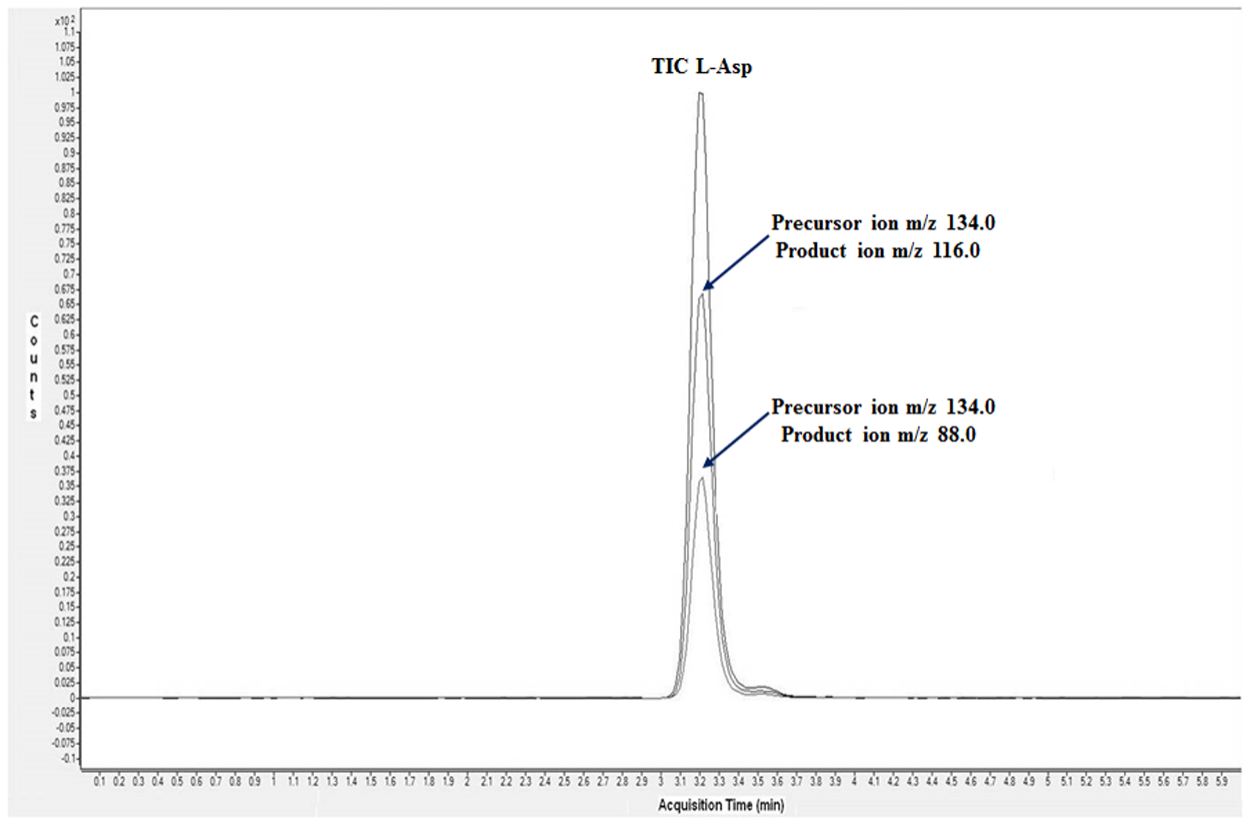
MRM chromatogram for a DDO treated matrix sample only displaying the l-Asp component. The specific MRM transitions for monitoring the l-Asp analyte are indicated.

Possible mass spectral interferences from biological specimens were then investigated. A control matrix only containing l-Asp was prepared by using a sample of adult mouse brain treated with DDO in order to completely remove endogenous d-Asp and NMDA [30]. The absence of any amount of free d-Asp in the sample used as control matrix was verified by LC-MS/MS analysis using the MRM method containing the transitions selected for the three analytes. As expected, Fig 3 shows the corresponding MRM chromatogram only displaying the l-Asp peak. No transitions for d-Asp and NMDA were detected demonstrating the absence of these metabolites in the control matrix.

### Method validation

**Fig 4:**
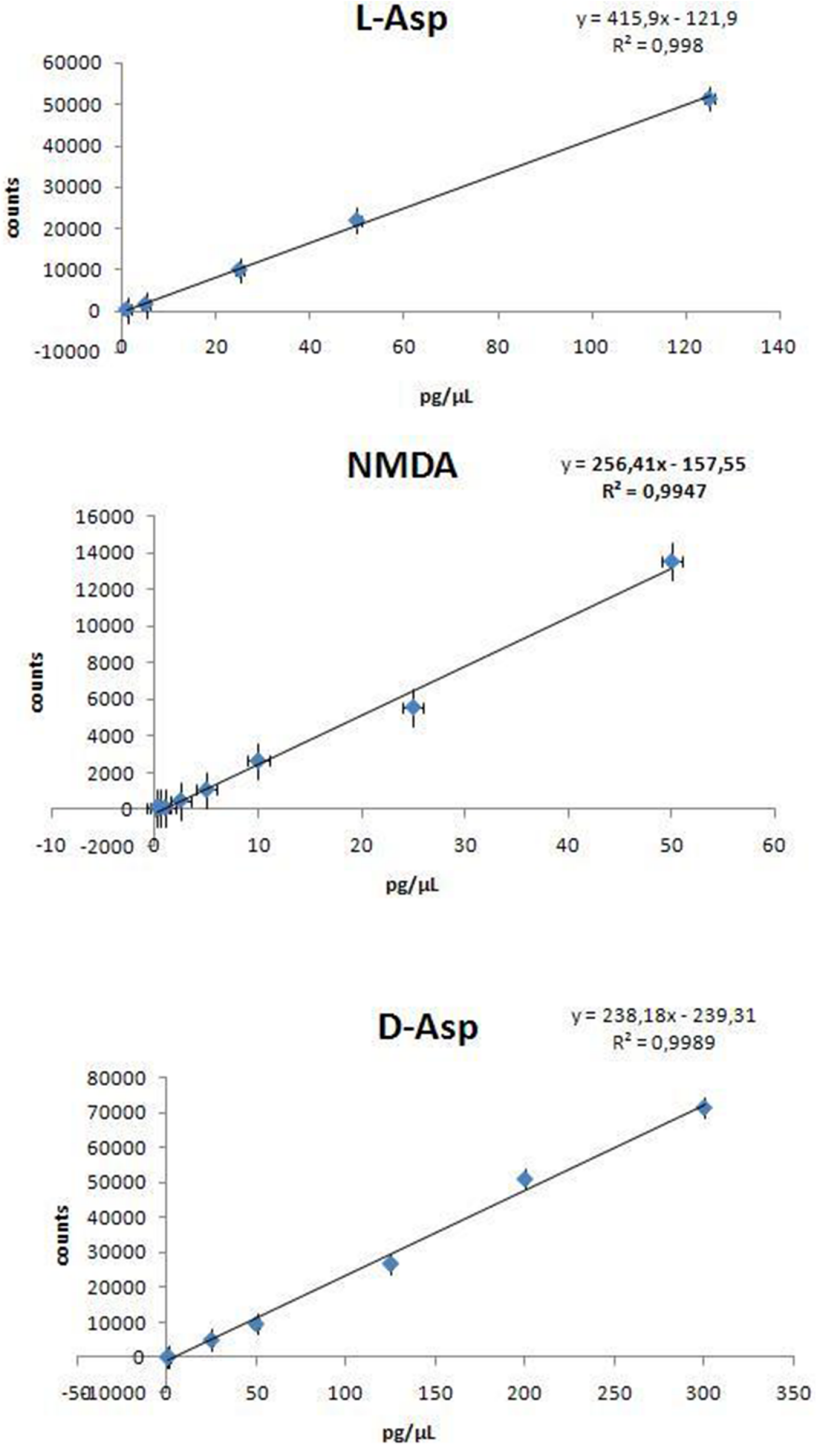
Calibration curves obtained for the three analytes.

Linearity and matrix effect were studied using standard solutions and matrix matched calibrations. Matrix matched calibration curves were prepared by spiking extracts from control tissues with known amounts of l-Asp, d-Asp and NMDA. Both standard and matrix matched calibration curves were constructed by plotting peak areas against concentration (pg/μl) and linear functions were applied to the calibration curves. Data were integrated by Mass Hunter quantitative software showing a linear trend in the calibration range for all molecules. The coefficients of determination (R2) were greater than 0.99 for all analytes. The signal corresponding to l-Asp was corrected for the presence of the endogenous metabolite in the control matrix. Fig 4 shows the standard calibration curves obtained for the three analytes.

The matrix effect was calculated as the percentage of the matrix matched calibration slope (B) divided by the standard calibration slope (A) yielding a matrix effect (B/A x 100) of 11–15% for the three analytes.

The limits of detection (LODs) were determined by making 10 replicate measurements of blank samples spiked with low concentrations of each analyte and calculated as: LOD = 3 * SD. The LODs of d-Asp, l-Asp and NMDA resulted to be 0.52 pg/μl for d-Asp, 0.46 pg/μl l-Asp and 0.54 pg/μl for NMDA, respectively.

The precision and accuracy at different concentration levels were calculated for each analyte and resulted to be in the range of %RSD = 1.0–9.0 with the recovery efficiency (%RE) being in the range 9.6–10.3, well suited for the quantitative measurement of these metabolites in brain tissues. Data were collected over a period of 10 days and each measurement represents the average of three experiments. Table 2 reports the determined parameters for l-Asp, d-Asp and NMDA.

**Table 2:** Validation parameters of the analytical method developed for the quantitative determination of l-Asp, d-Asp and NMDA. Each measurement represents the average of three technical replicates.

**NMDA**						
**Sample Conc. pg/μl**	**Response**			**Average**	**SD**	**RSD%**
	**1**	**2**	**3**			
0.25	101.4	92.4	108.0	100.6	7.8	7.8
2.5	481.0	433,5	510.6	475.0	38.9	8.2
5	1071.4	999.7	1137.4	1069.5	68.9	6.4
10	2649.2	2390.5	2825.5	2621.7	218.8	8.3
25	5579.2	5085.9	6054.0	5573.2	484.1	8.7
50	12956.4	11717.1	14243.7	12972.4	1263.3	9.7
l**-Asp**						
**Sample Conc. pg/μl**	**Response**			**Average**	**SD**	**RSD%**
	**1**	**2**	**3**			
1	300.2	288.4	296.3	295.0	6.0	2.0
5	1500.3	1425.6	1521.4	1482.4	50.3	3.4
25	9900.4	9852.1	9745.0	9832.5	79.5	0.8
50	21900.8	21105.3	22001.3	21669.1	490.8	2.3
125	51478.1	50421.4	51235.1	51044.9	553.5	1.1
d **-Asp**						
**Sample Conc. pg/μl**	**Response**			**Average**	**SD**	**RSD%**
	**1**	**2**	**3**			
1	50.7	54.88	45.5	50.3	4.7	9.4
5	82.4	75.60	87.4	81.8	5.9	7.2
25	309.3	323.70	282.1	305.0	21.1	6.9
50	579.3	620.46	625.9	608.6	25.5	4.2
80	823.6	876.54	866.1	855.4	28.0	3.3
125	1440.4	1526.87	1440.4	1469.2	49.9	3.4
**Compound**	**LOD (pg/μl)**	**LOQ (pg/μl)**	**Linear range (pg/μl)**	**Matrix effect %**		
d-Asp	0.52	1.57	1–125	11		
l-Asp	0.46	1.41	1–125	14		
NMDA	0.54	1.64	0.25–50	15		
**Compound**	**Linearity (R2)**		**Recovery ± RSD (%)**			
	**Standard**	**Matrix**	**50 pg/μl**	**100 pg/μl**	**200 pg/μl**	
d-Asp	0.998	0.996	73.9±1.1	87.8±9.4	106.8±1.3	
l-Asp	0.994	0.992	75.9±1.3	86.1±9.8	108.2±1.1	
NMDA	0.994	0.993	74.9±1.5	82.4±9.1	104.1±1.6	

In order to define the recovery in matrix and the LOQ, standard solutions were then prepared by spiking known amount of d-Asp, l-Asp and NMDA in the control mouse brain samples. Tissue samples were weighed and sonicated in TrisHCl 50 mM pH 8.0; the analytes were extracted and analysed by the LC-MS/MS procedure using the conditions previously defined. The endogenous background of each analyte was corrected in the Mass Hunter software by calculating the non-spiked signal. LOQ, calculated as the analyte concentration giving a signal to noise ratio (S/N) of 10, resulted to be 1.57 pg/μl for d-Asp, 1.41 pg/μl for l-Asp and 1.64 pg/μl for NMDA, respectively. Recovery was calculated by comparing the extraction yield before and after the addition of the different standard analytes into the control tissue samples. Quantitation was achieved by using the calibration curves. Recovery for each analyte was better than 90% (see Table 2).

### d-Asp, l-Asp and NMDA determination in mouse brain tissues

**Fig 5:**
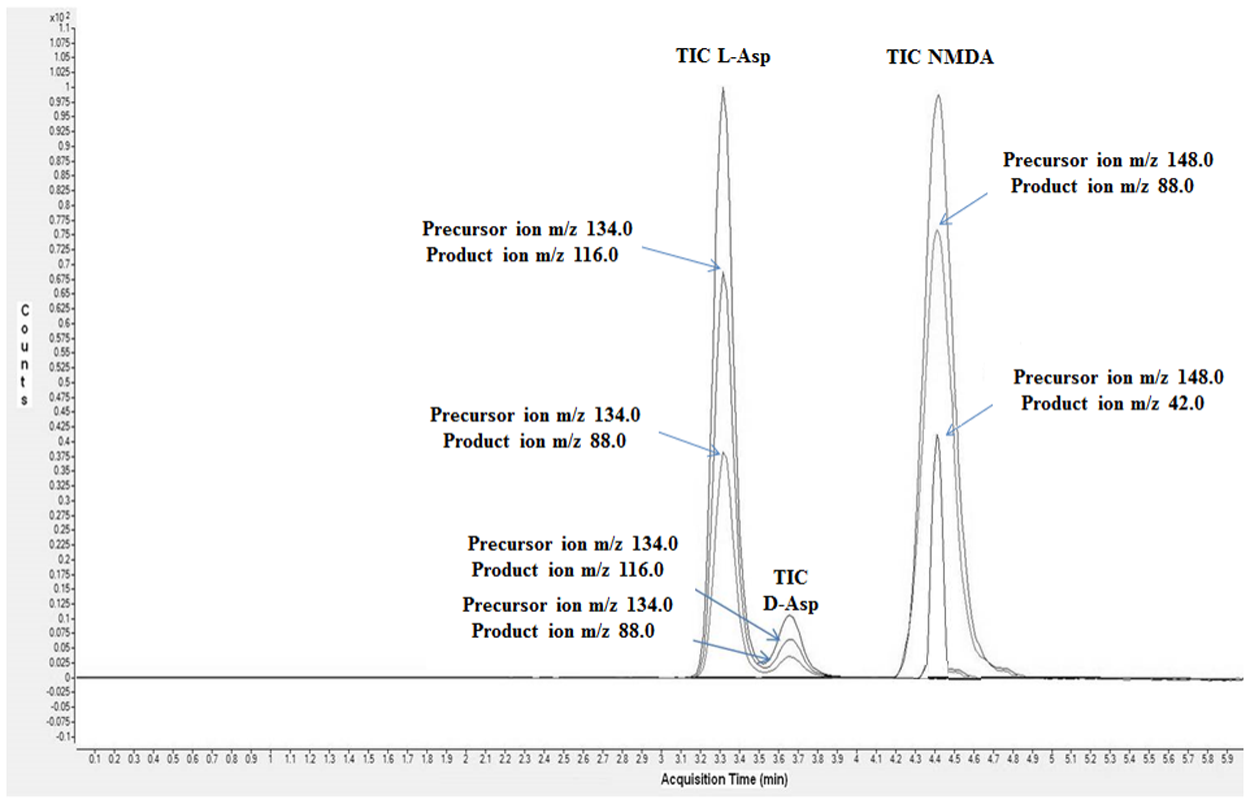
TIC and MRM chromatograms recorded for the three analytes in a sample from prefrontal cortex of wild type mouse. The specific MRM transitions for each analyte are indicated.

The reliability of the developed procedure and its possible application to clinical analyses were evaluated by determining the concentration of d-Asp, l-Asp and NMDA in several mouse brain tissues at different times of development. Freeze-dried specimens from mouse brain tissues were sonicated and precipitated according to the simple developed method, avoiding heavy sample handling. The supernatant from protein precipitation was directly analysed by tandem mass spectrometry in MRM scan mode. The analytes concentrations were calculated in pg/μl and then expressed in nmol/g of brain tissue in order to perform a comparison with data presented in the literature. As an example, Fig 5 shows the TIC and the MRM transitions recorded for the three molecules in a sample from prefrontal cortex of wild type mouse, indicating that the three metabolites could be easily quantitated.

**Fig 6:**
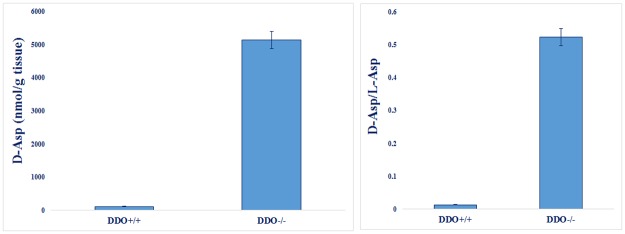
Quantitative analysis of d-Asp (left panel) and d-Asp versus l-Asp ratio (right panel) in samples from mouse prefrontal cortex using the developed LC-MS/MS MRM procedure. *DDO*^+/+^, wild type mouse; *DDO*^-/-^, d-aspartate oxidase (DDO) knockout mouse.

Finally, the same analysis was performed on prefrontal cortex samples from both wild type and knockout mice for *DDO* gene (*DDO*^+/+^ and *DDO*^-/-^, respectively) [30] (see Table 3). Fig 6 showed the results obtained by the LC-MS/MS procedure demonstrating that in *DDO*^-/-^ mice sample the d-Asp levels are much higher compared to *DDO*^+/+^ littermates. Moreover, the d-Asp/l-Asp ratio determined by LC-MS/MS (0.012 for *DDO*^-/-^ mice and 0.441 for *DDO*^+/+^, see Fig 6) was approximately coincident with those previously obtained by HPLC measurements [30] (0.019 for *DDO*^-/-^ mice and 0.624 for *DDO*^+/+^, manuscript in preparation).

**Table 3:** Quantitative determination of l-Asp, d-Asp and NMDA in samples from mouse prefrontal cortex by MRM LC-MSMS analysis. Samples were obtained from brain of three individual animals from each group (*DDO*^+/+^, wild type mouse; *DDO*^-/-^, D-aspartate oxidase (DDO) knockout mouse). Each measurement represents the average of three technical replicates.

Brain Tissue sample	l-ASP *nmol/g*	d-Asp *nmol/g*	NMDA *nmol/g*
Mouse 1 *DDO*^+/+^	8190±819	113±11	1.2 ±0.1
Mouse 2 *DDO*^-/-^	9830±983	5150±515	2.9±0.3
Mouse 3 *DDO*^+/+^	10600±1060	130±13	1.8±0.2
Mouse 4 *DDO*^-/-^	9990±990	5330±533	2.3 ±0.2
Mouse 5 *DDO*^+/+^	9340±934	127±13	1.5±0.1
Mouse 6 *DDO*^-/-^	9570±957	5230±523	2.4±0.2

## Discussion

Many *in vivo* and *in vitro* studies have suggested that d-Asp has a crucial role in NMDA receptor-mediated neurotransmission playing very important functions in physiological and pathological processes [12–14, 31]. Moreover, a significant reduction of d-Asp was observed in brain of patients with schizophrenia [13] in line with the hypothesis of a NMDARs hypofunction in this pathology [31]. These crucial biological functions trigger the need for a simple, reliable and effective methodology to quantitate the levels of d-Asp and its derivative NMDA in brain tissues, as the alteration of their amount would be of invaluable clinical and diagnostic value. As d-Asp occurs at very low concentration in most biological samples, a reliable extraction procedure with minimum sample handling needed to be also optimised.

Enantio-separation through liquid extraction technology is a well established field in key research areas such as pharmaceutical industry, food additives and life science [32–33]. Analytical methods using a chiral stationary-phase column [34] or pre-column derivatisation with a chiral reagent [35] have been developed for separation and determination of N-methyl-DL-aspartic acid (NMA) in bivalves, octopus and mollusk tissues. However, the use of derivatising agents is often not recommended due to the enantiomeric impurities and incomplete reactions giving false positive results and quantification errors. Moreover, LOD obtained for derivatised amino acids analysed by different HPLC methods resulted to be at least 10 times higher than the values obtained by MRM measurements [35–37]. As already demonstrated, the LOD for acidic amino acid by MS analysis was 100 times lower than that by HPLC-UV method and it was much lower for other amino acids [26]. The LOD values recorded by our MRM method resulted to be in perfect agreement, and even lower in some cases, with LODs measured in other LC-MS/MS analyses of d-amino acids in biological samples [38–41]. Comparable LOD of a few ppb was achieved also by coupling of fluorescence-based detection to chiral columns *e*.*g*. for d-Asp derivatised with 4-fluoro-7-nitro-2,1,3-benzoxadiazole quantified in rat brain tissues, plasma, and urine [42]. CE laser induced fluorescence (LIF) detection systems are increasingly used for the chiral separation of all the amino acids due to the high separation efficiency, and compatibility with sensitive detection methods including MS instruments [43]. Although the LOD by such method achieves concentration of the low nM for several molecules [44], recent data related to d-Asp quantification showed LOD of 17 nM (2.3 ppb) in chicks brain tissues [45] and 8 nM (1 ppb) in various brain regions of adult mice [46], 4 and 2 fold, respectively, higher than LOD recorded with our MRM method. Peak assignments for most of these methods needed to be confirmed [47].

In this paper, we describe a simple and rapid method for the simultaneous direct measurement of free d-Asp, L-Asp and NMDA in brain tissues based on chiral fractionation of enantiomers and tandem mass spectrometry in MRM mode avoiding any derivatisation step. The elution of extracted metabolites was accomplished using a mixture of organic solvent that provided an optimal coupling with mass spectrometry avoiding complex pre-cleaning of the samples and making the analytical procedure simpler and faster. d- and l-Asp were distinguished by their retention time on chiral chromatography and all three compounds could be unambiguously identified by their specific MRM transitions with LOD of 0.5 ppb. The MRM method was able to discriminate the target metabolites within the very complex mixture originated from prefrontal cortex samples of both *DDO*^+/+^ and *DDO*^-/-^mouse, an animal model in which the *DDO* gene has been genetically deleted [30]. These results were comparable with the same measurements carried out by HPLC [30] and the d-Asp/l-Asp ratio determined by LC-MS/MS was in very good agreement with that obtained by HPLC. The elevated amount of d-Asp in the knockout mouse was expected as the absence of DDO in the brain decreases the oxidation rate of d-Asp leading to an increase in concentration of the free amino acid. This event is thought to be correlated to schizophrenia. The enantiomer N-Methyl L-Asp (NMLA) was not included in the analyses as this metabolite is not involved in NMDA-receptor modulation and was demonstrated to be the less potent antagonist of Glu in brain synaptic membranes [48].

In conclusion, the developed method provided to be a reliable and effective procedure for direct measurements of free d-Asp, l-Asp and NMDA in brain tissues and was successfully applied as a rapid screening for detection of these metabolites levels in different mouse brain districts (manuscript in preparation). The relative simplicity of sample preparation together with its high sensitivity makes the LC-MS/MS MRM method well suited, not only for research work but also, for the measurement of clinical samples. The methodology we developed allows simultaneous d-Asp, l-Asp and NMDA profiling in brain but can be easily extended to different class of metabolites, and easily expandable to clinical samples screening for the follow-up of patients and/or to monitor therapeutic treatments.

## Supporting information

S1 Fig LC-MS/MS analysis in MRM mode of l-Asp, d-Asp and NMDA mixture. The TIC chromatogram and the specific MRM transitions for each analyte are indicated.
(PPTX)

S1 Table Experimental conditions for mouse brain sample treatment. Different extraction and precipitation conditions were tested and the percentage (%) of recovery of the three analytes averaged for three experiments for each pair of conditions were calculated. Conditions yielding the highest recovery for all three analytes were selected.
(PPTX)
