# Supplementary material for: Quantitative determination of free D-Asp, L-Asp and N-methyl-D-aspartate in mouse brain tissues by chiral separation and Multiple Reaction Monitoring tandem mass spectrometry
Source: PLoS One. 2017 Jun 29;12(6):e0179748. doi: 10.1371/journal.pone.0179748 (PMC5491048; doi:10.1371/journal.pone.0179748)
Supplement: S1 Fig — The TIC chromatogram and the specific MRM transitions for each analyte are indicated. (PPTX) [file pone.0179748.s001.pptx]

## Slide 1
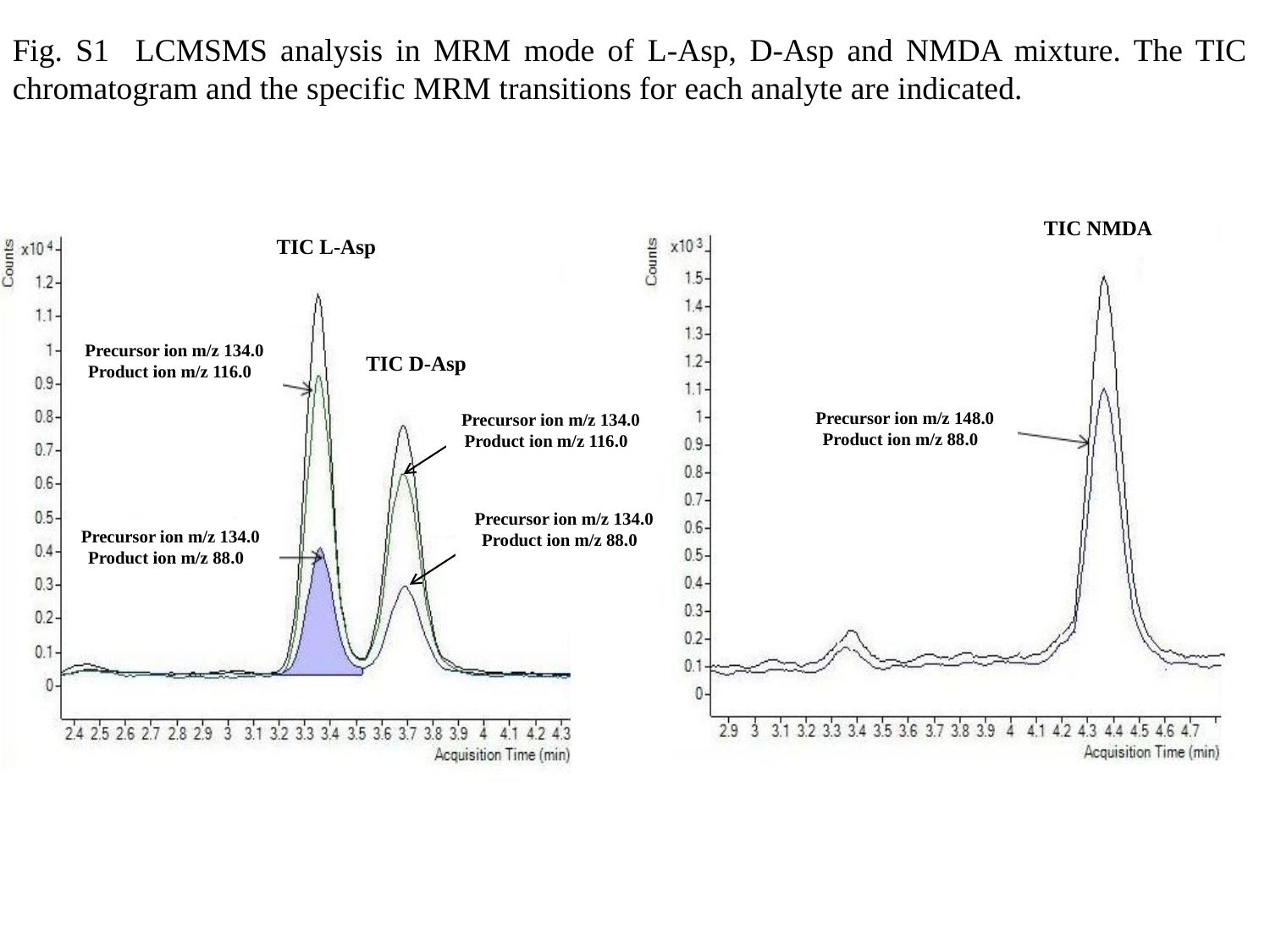

Fig. S1 LCMSMS analysis in MRM mode of l-Asp, d-Asp and NMDA mixture. The TIC chromatogram and the specific MRM transitions for each analyte are indicated.
TIC NMDA
TIC L-Asp
Precursor ion m/z 134.0
Product ion m/z 116.00
0
TIC D-Asp
Precursor ion m/z 148.0
Product ion m/z 88.00
00
Precursor ion m/z 134.0
Product ion m/z 116.00
0
Precursor ion m/z 134.0
Product ion m/z 88.00
0
Precursor ion m/z 134.0
Product ion m/z 88.00
0
