# Supplementary material for: Quantitative determination of free D-Asp, L-Asp and N-methyl-D-aspartate in mouse brain tissues by chiral separation and Multiple Reaction Monitoring tandem mass spectrometry
Source: PLoS One. 2017 Jun 29;12(6):e0179748. doi: 10.1371/journal.pone.0179748 (PMC5491048; doi:10.1371/journal.pone.0179748)
Supplement: S1 Table — Different extraction and precipitation conditions were tested and the percentage (%) of recovery of the three analytes averaged for three experiments for each pair of conditions were calculated. Conditions yielding the highest recovery for all three analytes were selected. (PPTX) [file pone.0179748.s002.pptx]

## Slide 1
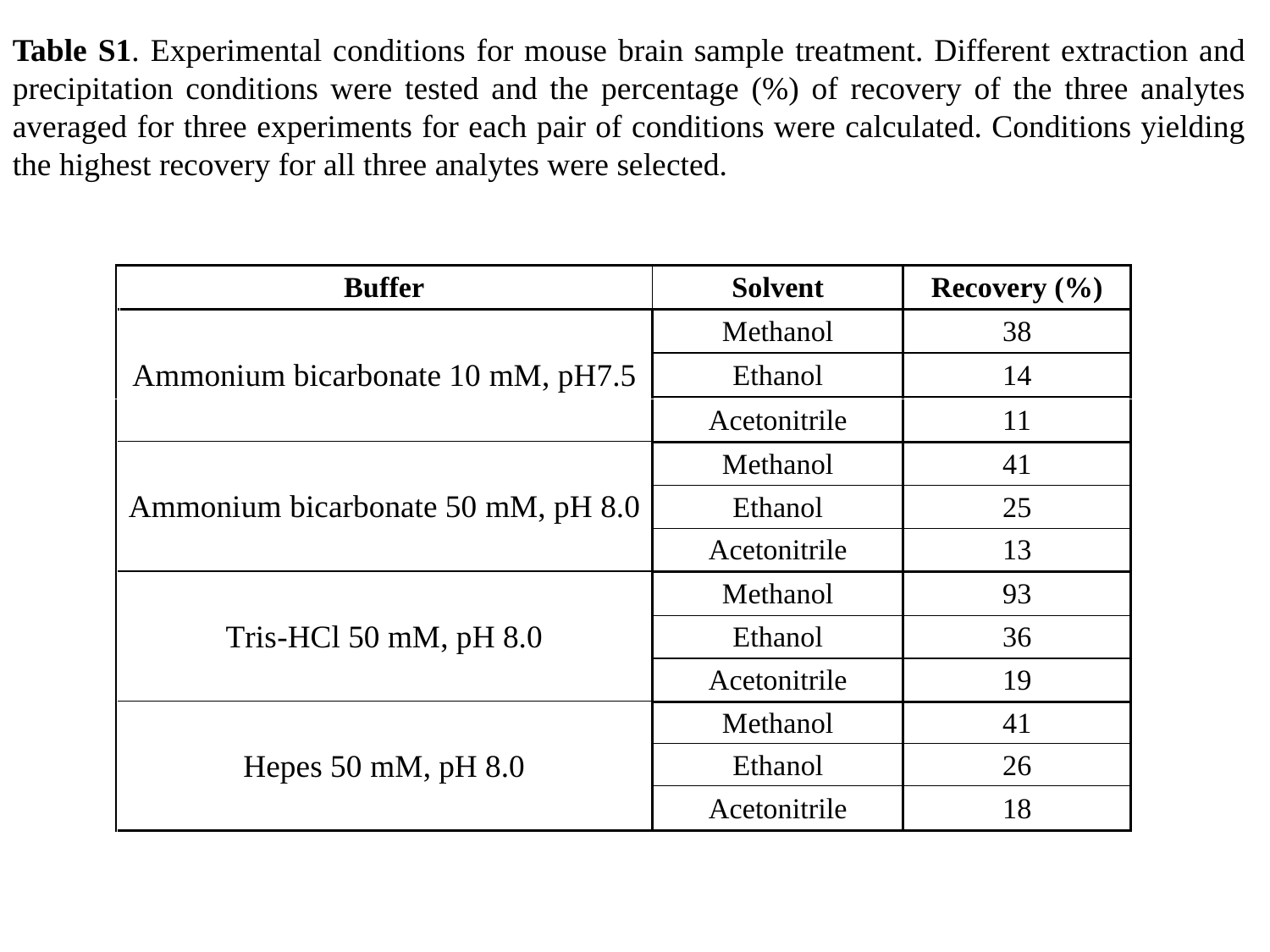

Table S1. Experimental conditions for mouse brain sample treatment. Different extraction and precipitation conditions were tested and the percentage (%) of recovery of the three analytes averaged for three experiments for each pair of conditions were calculated. Conditions yielding the highest recovery for all three analytes were selected.
